# Oral contraceptive use and anterior cruciate ligament injury: comparison of active comparator new user cohort and case-control study designs

**DOI:** 10.1186/s40621-020-00282-x

**Published:** 2020-10-19

**Authors:** Mackenzie M. Herzog, Jessica C. Young, Jennifer L. Lund, Virginia Pate, Christina D. Mack, Stephen W. Marshall

**Affiliations:** 1 Department of Epidemiology, Gillings School of Global Public Health, University of North Carolina at Chapel Hill, McGavran Greenberg Hall, CB #7435, Chapel Hill, NC 27599-7435 USA; 2 University of North Carolina Injury Prevention Research Center, Chapel HIll, USA; 3 Injury Surveillance and Analytics, Real-World Analytics Solutions, IQVIA, Durham, USA; 4 Department of Exercise and Sport Science, College of Arts and Sciences, University of North Carolina at Chapel Hill, Chapel Hill, USA

**Keywords:** Anterior cruciate ligament, Female, Knee injuries, Oral contraceptives

## Abstract

**Background:**

This study further investigates a protective association between oral contraceptive (OC) use and anterior cruciate ligament (ACL) injury noted in prior case-control studies.

**Methods:**

Active comparator new user cohort analysis of women aged 13–45 years in the United States from the IBM MarketScan Commercial Claims and Encounters database who newly-initiated low-dose OCs (exposed) or underwent intrauterine device (IUD) insertion (comparison group) from 2000 to 2014. Women were followed for ACL injury starting 90 days after OC initiation or IUD insertion until OC or IUD discontinuation or end of continuous enrollment. Adjusted hazard ratios (adjHR) and 95% confidence intervals (CI) were estimated controlling for age. Secondary analysis replicated previously-published case-control studies assessing “ever” versus “never” OC use over 1- and 5-year periods among women who underwent ACL reconstruction compared to age-matched controls.

**Results:**

In the cohort analysis, 2,370,286 women initiated OCs and 621,798 underwent IUD insertion. There were 3571 (0.15%) ACL injuries during an average 370.6 days of continuous OC use and 1620 (0.26%) during an average 590.5 days of IUD use. No difference in risk of ACL injury was observed between OC initiators and IUD users (adjHR = 0.95; 95%CI 0.89, 1.01). The case-control analysis replicated the slight protective association observed in prior studies over a 5-year period (OR = 0.90; 95%CI 0.85, 0.94).

**Conclusions:**

This cohort study suggests no association between OC use and ACL injury, while the case-control study suggested bias from uncontrolled confounding and selection factors may have influenced previous findings that suggested a protective association between OC use and ACL injury.

## Background

Anterior cruciate ligament (ACL) injuries are one of the most common and most significant knee injuries (Joseph et al. 2013; Gornitzky et al. 2015; Mall et al. 2014). While males sustain a larger absolute number of ACL injuries compared to females, studies have shown that females have higher incidence rates of ACL injury than males when accounting for participation in gender-comparable activities, such as basketball, soccer, and other collegiate sports (Brophy et al. 2015; Whitney et al. 2014; Lyle et al. 2014; Flaxman et al. 2014; Kosaka and Nakase 2016; Gould et al. 2016; Rahr-Wagner et al. 2014; Konopka et al. 2016; Bates et al. 2016; Arendt et al. 1999). Several risk factors have been proposed and investigated to explain the higher injury rates observed in females, including biological, biomechanical, and psychological factors (Kosaka and Nakase 2016; Gould et al. 2016; Rahr-Wagner et al. 2014; Konopka et al. 2016; Bates et al. 2016).

Multiple studies suggest that hormone levels may influence ACL injury risk in women (Hewett et al. 2007; Liu et al. 1996; Sarwar et al. 1996), and estrogen receptors, in particular, have been identified in the human ACL and have been hypothesized to potentially impact the synthesis of collagen, thereby potentially influencing ACL injury risk (Liu et al. 1996). Other studies have found a potential cyclic influence on risk of ACL injury among women, suggesting that fluctuation in serum estrogen level may affect ACL structure and composition (Hewett et al. 2007). Furthermore, biomechanical studies have identified increased muscle fatigue and knee laxity during the ovulation phase of the menstrual cycle, suggesting potential effects of hormone fluctuation on musculoskeletal and physiological performance (Sarwar et al. 1996).

Several prior studies have investigated the association between oral contraceptive (OC) use and ACL injury, under the working assumption that OC use might serve as a proxy for estrogen levels (Rahr-Wagner et al. 2014; Gray et al. 2015; Ruedl et al. 2009). Notably, two case-control studies reported a protective association between low-dose estrogen and progestin OC use (progestin only OCs excluded) and ACL injury (Rahr-Wagner et al. 2014; Gray et al. 2015). The largest of these, a study of 4497 cases and 8858 controls from the Danish Knee Ligament Reconstruction Registry found a decreased 5-year risk of ACL injury among OC users compared to non-users (Risk Ratio = 0.82; 95% CI 0.75–0.90) (Rahr-Wagner et al. 2014). However, these case-control studies raise methodologic concerns about the potential for uncontrolled confounding, inclusion of prevalent users resulting from “ever” versus “never” comparisons, and the long period of observation (up to 5 years) for OC use prior to the ACL injury. In particular, women who use contraception may be different (e.g. different activity level or health-seeking behaviors) from those who do not leading to confounding by indication (Kyriacou and Lewis 2016). Furthermore, assessment of women who had at least one prescription (“ever” users) are a heterogeneous group consisting of those who initiated and continuously used an OC, those who only received one or multiple sporadic prescriptions, and women who used OCs historically but discontinued use prior to the outcome of interest. For example, a woman who had one OC prescription 5 years prior to the ACL injury would be considered to have the same exposure to estrogen as a woman who took OCs monthly for 5 years prior to the ACL injury.

An active comparator new user study design selects patients who have a similar health indication (eg. need for systemic birth control), but are exposed to different treatment options (oral contraception vs IUD) (Lund et al. 2015). These patients are expected to have similar baseline health indications, health seeking behavior, severity of symptoms, etc., thus the impact of the exposure of interest (OC) can be more easily assessed. This study design minimizes baseline confounding and improves study validity (Kyriacou and Lewis 2016). Another key strength of the active comparator new user study design is the ability to synchronize the start of follow-up by focusing on new users of the treatment options, which helps to avoid issues with time-varying hazards that may result from inclusion of prevalent users. We therefore sought to further investigate this association in a large cohort of commercially-insured US women. Our study aims were to 1) quantify the association between OC use and ACL injury using an active comparator new user study design, and 2) compare the results of the active comparator new user study design to results obtained by replicating a “ever” vs. “never” OC use case-control study in the same population. In our active comparator new user design, the OC “new user” group (“exposed”) was compared to women who underwent an IUD insertion, a different therapeutic intervention commonly used for the same indication, rather than the entire population of non-OC users.

## Methods

The 2000–2014 data from the IBM MarketScan Commercial Claims and Encounters database, an administrative database that contains healthcare utilization information for a large sample of commercially-insured individuals, was used for this analysis. The database contains over 20 billion claims for approximately 158 million active employees and their dependents, early retirees, and COBRA beneficiaries who are covered by employer-sponsored private health insurance in the United States (Commercial Claims and Encounters Medicare Supplemental SOURCE 2013). The database includes eligibility information for individuals enrolled in employer-based health insurance and medical and drug claims provided by multiple employers and health plans who have agreed to participate (IBM 2019). Because the database is generated based on employer-based health insurance rather than specific insurance carriers, individuals are included in the database if they switch insurance, provided the new insurance information is submitted by the employer or health plan. The study was determined to be exempt from review by the *[blinded for review]* institutional review board.

### Cohort study design

The exposure of interest was low-dose OC use; high- and middle-dose formulations are no longer recommended due to potential side effects such as venous thromboembolism (Russell and Ramcharan 1987), and these have been excluded from the analysis to align with prior research on this topic (Rahr-Wagner et al. 2014). As noted above, we used an active comparator group, new IUD insertion, to reduce potential confounding by indication (Lund et al. 2015). All women aged 13–45 years who newly-initiated low-dose OCs (“exposed”) or underwent intrauterine device (IUD) insertion (“comparator”) between 2000 and 2014 were identified from the database. To ensure complete capture of baseline health status and medication use, we required continuous enrollment in a health insurance plan included in the database including use of the prescription benefit in the past 180 days prior to initiation of low-dose OC or IUD placement. Women with prior use of OCs, evidence of IUD placement or removal, or history of prior ACL injury during the 180-day “washout period” prior to the index exposure were excluded in order to identify new OC and IUD users and incident ACL injuries.

**Fig. 1:**
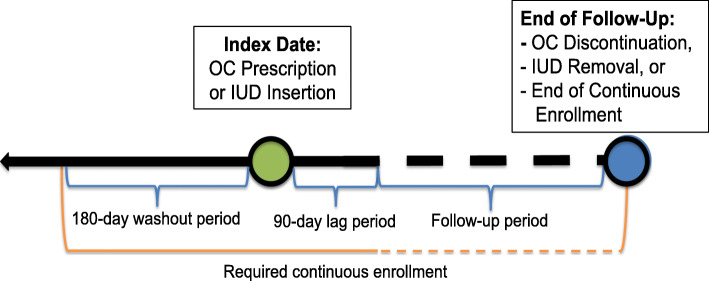
Study schematic of inclusion criteria for the active comparator new user cohort study

Low-dose OC initiators were identified using a crosswalk between Anatomical Therapeutic Chemical (ATC) codes to National Drug Codes (NDCs). Consistent with previous studies, ATC codes G03AA and G03AB were included, which include oral contraceptive combinations of estrogen and progestin (Rahr-Wagner et al. 2014). Low-dose OCs were defined as formulations containing ≤35mcg ethinyl estradiol (Rahr-Wagner et al. 2014). NDC codes included within ATC G03AA and G03AB that contained >35mcg ethinyl estradiol were excluded. Progestin only oral contraceptives (ATC G03AC) were excluded. Initiation on a low-dose oral contraceptive was considered the first prescription after a 180-day washout period with no evidence of OC prescription fills or IUD placement/removal (Fig. 1). Insertion of an IUD was identified in physician claims using the Current Procedural Terminology (CPT) code 58300 or International Classification of Diseases, Clinical Modification, 9th Edition, (ICD-9-CM) procedure codes 69.7X.

To allow for a period of hormonal stabilization following OC initiation, women were followed for ACL injury starting 90 days after OC initiation or IUD insertion until the earliest of the following events: 1.) ACL injury, 2.) OC discontinuation (defined using days’ supply + a 30-day grace period), 3.) IUD removal, 4.) switching to an alternative contraceptive method from the method initiated, 5.) end of continuous enrollment (e.g. individual no longer covered by an insurance plan included in the database), or 6.) end of the study period (December 31, 2014; Fig. 1). Individuals who were no longer covered by an insurance plan included in the database were censored at the time of disenrollment. ACL injury was defined as presence of an ICD-9-CM diagnosis code 717.83 or 844.2 or a CPT code 29888 for ACL reconstruction.

### Replication of prior case-control design and analyses

For the secondary aim, we replicated the prior case-control study designs to compare the results to the active comparator new user cohort among the same population. Women aged 13–45 years old who underwent incident ACL reconstruction (identified by CPT code 29888) were identified as cases for the case-control study. Women were required to have at least 360 days of continuous enrollment immediately prior to reconstruction to washout for evidence of prior ACL injury. As with the cohort design, women with no evidence of use of their prescription benefits during the washout period were excluded. Each ACL reconstruction case was randomly matched with replacement to two controls (women with no evidence of ACL reconstruction) who met the same inclusion criteria using an incidence density sampling method with exact matching based on age at time of surgery. Controls were assigned an index date identical to the case ACL reconstruction date.

**Fig. 2:**
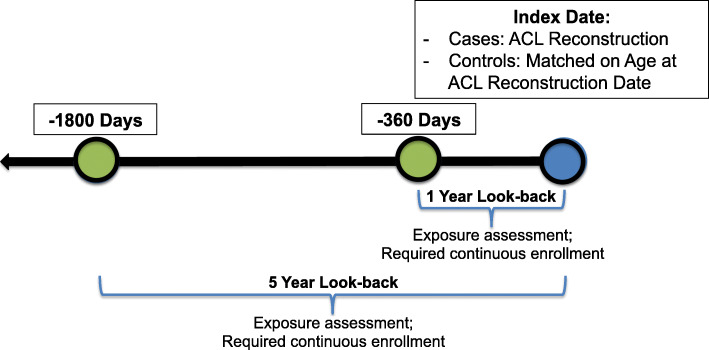
Study schematic of inclusion criteria for the “ever” versus “never” user case-control study replication

Analyses replicating the previously published case-control study design were implemented in this population comparing one or more claim for low-dose OC (“ever” users) compared to no OC claims (“never” users”) in the 1- and 5-years prior to ACL reconstruction (Fig. 2). Low-dose OC use was defined using the same criteria as the primary analysis.

### Statistical analysis

Descriptive statistics were calculated for relevant and available baseline characteristics of the study population, including mean age with standard deviation and range. For the cohort analysis, a crude Cox proportional hazard model was calculated to estimate a hazard ratio (HR) and 95% confidence intervals (CI). Adjusted HRs were estimated using a weighted Cox proportional hazard model after standardizing to the overall cohort age distribution using inverse probability of treatment weighting.

For the case control analysis, an odds ratio (OR) and 95% CI were estimated using conditional logistic regression, adjusting for age differences between cases and controls using 2:1 individual-level matching by age. The use of an incidence density sampling strategy was used to select controls from those at risk each time a case occurs, resulting in the OR approximating an incidence rate ratio. The participants in the case control analysis (Aim 2) were not identical to the participants in the cohort analysis (Aim 1), although some overlap occurred.

## Results

### Cohort study

There were 2,370,286 women who initiated low-dose OCs and 621,798 who underwent IUD insertion during the study period. Women initiating OCs were slightly younger than women undergoing IUD insertion and the average length of follow-up was slightly longer among IUD users (Table 1).

**Table 1:** Participants included in the active comparator new user cohort study, IBM MarketScan Commercial Claims and Encounters Database, 2000–2014

	OC Initiators ***n*** = 2,370,286		IUD Users ***n*** = 621,798	
	Mean (SD)	Range	Mean (SD)	Range
**Age (years)**	26.7 (8.1)	13, 45	32.4 (6.8)	13, 45
**Follow-up (years)**	1.1 (1.0)	0.2, 14.2	1.6 (1.5)	0.2, 14.5

**Fig. 3:**
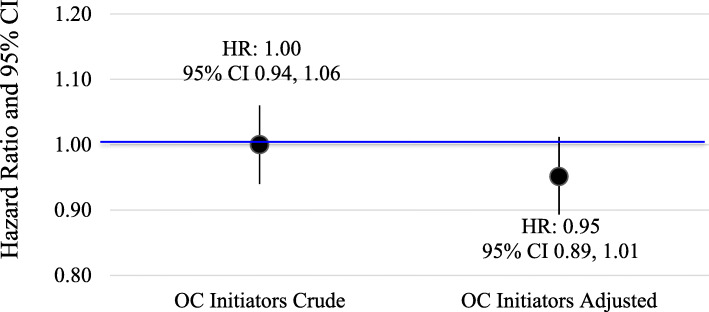
Crude and adjusted hazard ratios comparing OC initiators to IUD users in the active comparator new user cohort study, IBM MarketScan Commercial Claims and Encounters Database, 2000–2014

There were 3571 (0.15%) ACL injuries among women with continuous OC use and 1620 ACL injuries (0.26%) among women with continuous IUD use. Overall, there was no difference in crude risk of ACL injury comparing OC initiators to IUD users (Fig. 3; HR = 1.00, 95% CI 0.94, 1.06). The lack of association between OC initiation and risk of ACL injury persisted after adjusting for the age difference between groups, although there was a slight shift in the point estimate in a protective direction (Fig. 3; adjHR = 0.95; 95% CI 0.89, 1.01).

### Replication of prior case-control design and analyses

**Fig. 4:**
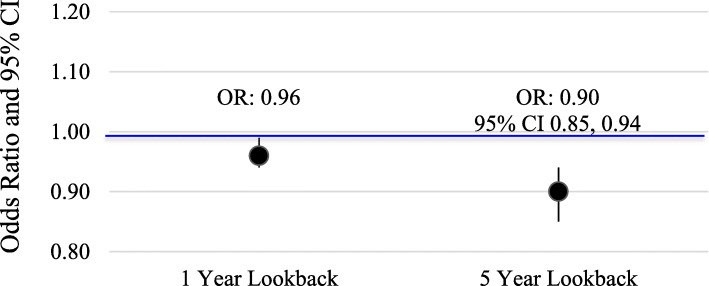
Odds ratios comparing “ever” vs. “never” OC use in the 1- and 5-years prior to ACL reconstruction for cases and age-matched controls in the case-control study replication, IBM MarketScan Commercial Claims and Encounters Database, 2000–2014

There were 50,215 ACL reconstruction cases that met the inclusion criteria for the 1-year look-back period and 100,429 age-matched controls (Table 2). Among the cases, 9772 (19.5%) had at least one prescription for OCs during the 1-year look-back period and were considered “ever” OC users. In comparison, 20,149 (20.0%) controls were “ever” OC users in the 1-year look-back. The odds of ACL reconstruction were 4% lower among “ever” OC users compare to “never” OC users (Fig. 4; OR = 0.96, 95% CI 0.94, 0.99).

**Table 2:** Age of participants included in the “ever” versus “never” case-control study replication, IBM MarketScan Commercial Claims and Encounters Database, 2000–2014

1 Year Lookback	**Cases** ***n*** **= 50,215**		**Controls** ***n*** **= 100,429**	
	**Mean (SD)**	**Range**	**Mean (SD)**	**Range**
	25.6 (10.4)	13, 45	25.6 (10.4)	13, 45
5 Year Lookback	**Cases** ***n*** **= 11,022**		**Controls** ***n*** **= 22,044**	
	**Mean (SD)**	**Range**	**Mean (SD)**	**Range**
	23.9 (10.4)	13, 45	23.9 (10.4)	13, 45

For the 5-year look-back period in the case-control study, we identified 11,022 cases and 22,044 age-matched controls meeting inclusion criteria. Controls were more likely to have had at least one prescription for OCs (*n* = 6548, 29.7%) during the 5-year look-back period compared to cases (*n* = 3032, 27.5%), resulting in 10% lower odds of ACL reconstruction among “ever” OC users compared to “never” OC users (Fig. 4; OR = 0.90, 95% CI 0.85, 0.94).

## Discussion

Results from the active comparator new user cohort study suggested a lack of association between OC use and ACL injury. In contrast, while the difference between point estimates from the cohort and case control analyses was minimal (adjHR = 0.95 vs. OR = 0.90, respectively), results replicating a case-control design that compares OC users to non-users over 5 years but may be vulnerable to bias reproduced the apparent slight protective association between OC use and ACL injury observed in prior studies. These findings suggest that the protective results from the prior case-control designs could be due to bias and support the use of active comparator new user cohort designs to help reduce bias in observational studies using administrative databases (Lund et al. 2015; Ray 2003; Hernan et al. 2008).

The principal strengths of the active comparator design stem from the ability to synchronize the start of follow-up and avoid issues with time-varying hazards that may result from inclusion of prevalent users, as well as reduce potential confounding by indication that could result when comparing “ever” vs. “never” users. In particular, women who use OCs may be more likely to use reversible contraception during their reproductive years shortly prior to starting a family or may be more likely to use OCs following a prior pregnancy, both of which may influence physical activity level. While controlling for age may serve as a proxy for history of pregnancy or future reproduction, it is likely that confounding may remain between “ever” and “never” OC users. Aside from differences in reproductive life stages or intentions, women who use OCs may be more likely to practice other health-conscious behaviors (e.g., participation in injury prevention activities, regular physical conditioning, etc.) than women who do not use OCs, which could also influence risk of ACL injury. Although not a perfectly exchangeable comparator group, use of women undergoing IUD insertion as an active comparator may help reduce potential for such unmeasured confounding between groups as compared to the prior case-control studies.

An additional strength of this study is its more precise measurement of timing between initiation of OC use and onset of ACL injury. The use of OCs as a proxy for hormone levels requires consideration of the time period over which OCs may reasonably influence hormone levels. If an association between OCs and ACL injury exists, we would expect this association to be confined to a period when OCs could reasonably contribute to hormone levels and hormonal stability, which is likely during or immediately following continuous OC use. The active comparator new user cohort design lends itself to improved assessment of this association with the clearly defined temporality from inclusion of OC initiators and periods of continuous use with a short grace period (30 days) between and following prescription fills. For this study, we also used a 90-day lag period prior to assessing women for ACL injury to allow for a period of hormonal stabilization following OC initiation. In contrast, assessment of “ever” OC users within a relatively long period of time (up to 5 years) mixes women who continuously used OCs over the exposure assessment period with women who received only one prescription and women who used OCs historically but discontinued use prior to the ACL injury.

### Limitations

As with previous studies on this topic, results from this study of low-dose OCs may not be generalizable to high- or middle-dose OCs. Based on our inclusion criteria, there is also a small potential for misclassification of new OC users if women used high- or middle-dose OCs during the washout period. This study also restricted to women who use combined estrogen and progestin OCs. Subsequently, results are not generalizable to those who use progestin only OCs. It is also important to note that both hormonal and non-hormonal IUDs were included in the comparator group for the cohort study, which sought to assess the impact of estrogen on ACL injury. It is possible that progestin may independently influence ACL injury risk, which was not assessed in this analysis. In addition, it is possible that OC use may influence ACL injury risk over a different time period. As noted above, this study assessed continuous OC use and started following women for the outcome 90 days after initiation. We followed women through continuous use; however, it is possible that OCs may have an impact on hormone levels over a different period of time. For example, women may require a time period different from 90 days to establish hormonal stability following initiation on an OC, or hormonal effects of OCs may continue longer than 30 days following discontinuation and therefore influence ACL injury risk over a different time period. It is also possible that women without continuous insurance enrollment are meaningfully different from those who have continuous insurance enrollment and were included here, and results may not be generalizable to those women. Finally, as mentioned above, unmeasured confounding may remain between women who use low-dose OC and women who undergo IUD insertion, including activity level or health-seeking behaviors. Previous studies on this topic controlled for variables such as age, immigration, income, pregnancies and births, proxies for physical activity level including clinically diagnosed obesity, prior lower extremity injury, prescription use of non-steroidal anti-inflammatories, and comorbid conditions such as asthma, diabetes mellitus, and infection (Rahr-Wagner et al. 2014; Gray et al. 2015), which we did not measure or control for in this analysis. Use of an active comparator (as opposed to comparison group of all non-users) improves validity by reducing the potential for confounding between groups, but neither comparator group assessed in this analysis (the IUD group for the cohort study or non-users in the case-control study) provide a perfect exchangeability. Theoretically, there should be very few potential confounders that influence a woman’s choice of contraception and also influence the risk of ACL injury; however, it is possible that confounding remains between our OC-exposed and active comparator (IUD) groups.

## Conclusions

This study suggests no association between oral contraceptive use and ACL injury. Bias from uncontrolled confounding and selection factors may have influenced previous findings from case-control designs that suggested a protective association between OC use and ACL injury.

## Data Availability

The data that support the findings of this study are available from IBM but restrictions apply to the availability of these data, which were used under license for the current study, and so are not publicly available. Data are however available from the authors upon reasonable request and with permission of IBM.
